# Rate of Perceived Exertion Based on Repetitions in Reserve Versus Percentage of One-Repetition Maximum for Resistance Training Prescription in Cardiac Rehabilitation: A Pilot Study

**DOI:** 10.3390/jcdd12010008

**Published:** 2024-12-27

**Authors:** Alessandro Gismondi, Ferdinando Iellamo, Giuseppe Caminiti, Barbara Sposato, Emanuele Gregorace, Valentino D’Antoni, Deborah Di Biasio, Sara Vadalà, Alessio Franchini, Annalisa Mancuso, Valentina Morsella, Maurizio Volterrani

**Affiliations:** 1 Cardiopulmonary Department, IRCCS San Raffaele, 00163 Rome, Italy; alessandro.gismondi@sanraffaele.it (A.G.); barbara.sposato@sanraffaele.it (B.S.); valentino.dantoni@sanraffaele.it (V.D.); debbydibiasio20@gmail.com (D.D.B.); vada.sara21@gmail.com (S.V.); alessio.franchini@sanraffaele.it (A.F.); annalisa.mancuso@sanraffaele.it (A.M.); valentina.morsella@sanraffaele.it (V.M.); maurizio.volterrani@sanraffaele.it (M.V.); 2 Department of Clinical Sciences and Translational Medicine, Tor Vergata University, 00133 Rome, Italy; iellamo@uniroma2.it (F.I.); emanuele.gregorace@gmail.com (E.G.); 3 Department of Human Science and Promotion of Quality of Life, San Raffaele Open University, 00163 Rome, Italy

**Keywords:** cardiac rehabilitation, exercise prescription, resistance training, RPE, muscular strength

## Abstract

The aims of this study were to assess the efficacy of the rate of perceived exertion (RPE) scale based on the number of repetitions in reserve (RIR) before exhaustion for the prescription of resistance training in cardiac rehabilitation and to compare it to the percentage of estimated one-repetition maximum (1RM) prescription method. Sixteen male patients (age 60 ± 8) with history of coronary artery disease were randomly assigned to two resistance training rehabilitation protocols lasting nine weeks and consisting of three sessions per week, with the same exercise selection, number of sets and repetitions, and rest periods, but different load prescription method (RPE vs. %1RM). Patients’ strength was evaluated pre- and post-intervention. Patients in the RPE group showed significant increases in strength across all the exercises of the protocol (leg press 24.25 ± 17.07 kg; chest press 7.25 ± 3.41 kg; seated row 13.88 ± 7.57 kg; leg extension 14.24 ± 4.53 kg; shoulder press 5.75 ± 4.06 kg; lat pulldown 7.50 ± 4.66 kg). Post-intervention between-group analysis showed no differences in strength gains (leg press *p* = 0.955; chest press *p* = 0.965; seated row *p* = 0.763; leg extension *p* = 0.565; shoulder press *p* = 0.868; lat pulldown *p* = 0.780) and trivial effect sizes (ES) for one prescription method over the other (leg press ES = −0.03; chest press ES = 0.00; seated row ES = 0.10; leg extension ES = −0.29; shoulder press ES = 0.18; lat pulldown ES = 0.05). RPE based on RIR seems to be an effective prescription method for resistance training in cardiac rehabilitation, showing similar efficacy to the standardized practice of percentage of 1RM.

## 1. Introduction

Cardiac rehabilitation (CR) is a secondary prevention multidisciplinary intervention [1] recommended by current clinical practice guidelines for the long-term management of patients with coronary artery disease (CAD) [2,3]. The central component of CR is exercise training, currently recommended as class I level of evidence A intervention for its effectiveness in improving cardiovascular (CV) risk profile and quality of life (QoL) and reducing hospitalizations [4], even in the modern era of acute revascularization and statin therapy [5].

However, in the last years, multiple lines of evidence have questioned the real efficacy of the exercise-based CR when compared to optimal medical treatment with no exercise for important primary endpoints such as recurrent CV events, CV and all-cause mortality [6,7,8]. Given the absence of international consensus on exercise prescription for CR [9], the large heterogeneity in the formulation of training used in clinical trials [10], and the lack of detailed description in reporting training protocols [11], no definitive conclusion can be drawn based on the current scientific literature. In fact, underdosed exercise does not lead to improvements in fitness since the imposed demands of the training stimulus must be sufficiently high to elicit the desired adaptations [12]. Hence, erroneous prescription could be the primary reason for the poor effectiveness of the exercise-based CR [13].

Regarding the modality of exercise used in CR, recently there has been growing interest in resistance training (RT), since over the last decade evidence has been accumulating about its benefit for CR [14]. Muscle strength is in fact a strong predictive factor for CV, cancer, and all-cause mortality both in healthy [15] and CAD patients [16] and a recent meta-analysis showed that adding RT to CR leads to greater improvements in physical fitness and muscle strength in CAD patients [17]. In fact, current recommendation for exercise in CR are to perform a combination of both aerobic and resistance training [18].

If the primary aim of including RT in CR is to improve muscle strength, then an appropriate dose of training should be prescribed, with recent evidence now suggesting that high intensity RT should be preferably administered [19,20]. Moreover, when compared to low intensity–high volume, high intensity RT has been proved to be more effective in increasing strength while being safe and holding less CV demands [21]. If high intensity RT promotes greater strength gains and it is safe to use in clinical setting, the most relevant question practitioners should ask is how to accurately prescribe it. Previously, the rate of perceived exertion (RPE) was validated as effective and practical method for prescribing and monitoring aerobic exercise in CR [22] and recently a new RPE scale based on number of repetitions in reserve (RIR) was developed [23] and subsequently validated in the literature as effective training tool for RT [24]. Compared to percentage of one-repetition maximum (1RM), RPE based on RIR load assignment has been proven to be equally effective or superior in increasing muscle strength and hypertrophy [25,26]. This subjective method of prescription can be particularly useful for autoregulation and individualization purposes [27], both important training factors to consider, especially in clinical settings with frail patients, and it has never been studied in CR.

Therefore, the purposes of this study were (a) to assess the efficacy in terms of strength adaptations of the RPE based on RIR for the prescription of RT in CAD patients and (b) to compare it to the percentage of 1RM-based prescription. We hypothesized that the novel RPE scale would be an effective method for prescribing RT in CR and that it would provide similar strength gains compared to the consolidated practice of percentage-based prescription.

## 2. Materials and Methods

### 2.1. Population

A total of twenty-one male patients with history of CAD were recruited between October 2022 and June 2023 at the San Raffaele IRCCS rehabilitation facility in Rome. After the recruitment, two patients developed contraindications to exercise training and three were excluded due to non-adherence to the rehabilitation protocols (RPs); therefore, only sixteen patients completed the RPs (eight per group). Inclusion criteria for the patients were history of CAD, clinical stability with no hospital admissions for heart failure (HF) in the previous six months or modifications to pharmacological therapy in the previous three months, and New York Heart Association (NYAH) functional class I. Patients would be excluded if they had unstable angina, uncontrolled hypertension or arrhythmias, exercise test positive for myocardial ischemia or complex arrhythmias, symptomatic valvular or peripheral arterial disease, severe chronic obstructive pulmonary disease, neurologic or orthopedic conditions contraindicating or limiting exercise training.

### 2.2. Study Design

This study was conducted as a prospective longitudinal randomized trial. Patients were initially evaluated in the screening visit, during which inclusion and exclusion criteria were examined. The evaluations included clinical history collection, echocardiography and ergometric test. All these investigations were carried out as part of the routine approach to patients before starting RPs. Patients who met the criteria were then asked to participate in this study. Patients were randomly assigned to two RT RPs with the same exercise selection, sets, repetitions, and rest periods, but different load prescription method: one group using RPE based on RIR (RPE group) and the other using percentage of 1RM (%1RM group). A randomization code was developed by random number generator software to ensure the randomness of the assignment. The RPs lasted nine weeks with three training sessions per week following a linear periodization model, with exercises involving the major muscle group of the upper and the lower body. A detailed description of the RPs according to the Consensus on Exercise Reporting Template (CERT) [28] is illustrated in Section 2.5. This study complied with the Declaration of Helsinki and was approved by the local Ethics Committee of IRCCS San Raffaele Roma (protocol number 13/2022). All patients gave written informed consent before participating in this study.

### 2.3. Cardiologic Evaluation

Prior the administration of the RPs, all patients underwent a full clinical evaluation consisting of complete medical history, cardiac and chest physical examination, anthropometric measurements, transthoracic echocardiogram (Vivid E95, GE Healthcare, Chicago, IL, USA), and maximal exercise test performed on a cycle ergometer (Mortara Instrument, Casalecchio Di Reno, Italy) with a 20 × 2 incremental protocol (20-Watt increases every 2 min). Patients were required to exercise at a speed of 65 revolutions per minute (RPM) and the test was terminated when the RPM could not be maintained despite increasing effort.

### 2.4. Strength Testing

Patients’ strength was evaluated by a Certified Strength and Conditioning Specialist using six repetitions maximum (6RM) tests on all the exercises of the RP according to validated procedures [29]. This type of test was chosen because it closely matches the repetition scheme used in the RPs (see more in the dedicated section), hence could accurately detect strength changes in this repetition range.

In preparation for the test, the warm-up consisted of one set of ten repetitions with minimal or no load, followed by sets of six repetitions with low load. Weight was then progressively increased from set to set until reaching momentary failure during the sixth repetition, i.e., the inability to complete the concentric phase of the lift despite maximal effort, and the weight used in the previous set was recorded as the 6RM. Even though current recommendation suggests that patients can avoid the Valsalva maneuver with breathing technique, participants were allowed to use it during the tests, since previous studies demonstrated that it is reflexively activated at high intensities and close to muscle failure [30]; therefore, advising against it would have altered the results of the tests.

During the tests, the RPE scale based on RIR was shown to the patients, along with verbal aid on how to determine scores. The scale goes from 1 to 10 based on the subjective determination of RIR prior reaching momentary failure, so that a score of 10 indicates the maximum number of repetitions for a given load could be performed, 9 indicates only one more repetition could be performed, 8 indicates only two more repetitions could be performed, etc., as shown in Table 1.

The 6RM tests were performed with Biocircuit Wellness System machines (Technogym, Cesena, Italy) on two separate occasions 72 h apart to accurately determine patients’ strength by avoiding excessive fatigue of multiple tests and to anchor RPE scores to the perceived fatigue and the maximal effort. The first strength test session consisted of leg press, chest press, and seated row, while the second consisted of leg extension, shoulder press, and lat pulldown. Based on previously reported relationship between percentage of 1RM and multiple repetitions completed [22], the 6RM results were used to estimate the 1RMs to then calculate the percentage of load for the RP.

### 2.5. Rehabilitation Protocol

The RPs consisted of nine weeks of RT exercise, as shown in Table 2. Both groups trained three times per week on non-consecutive days (Monday, Wednesday, and Friday), using the same exercises, sets, repetitions, and rest periods. The only difference between the two RPs was that in the %1RM group the loads were selected as percentage of the estimated 1RMs (e1RMs) based upon the pre-intervention strength tests, while in the RPE group the patients self-selected the loads to reach the RPE targets. RPE scores of both groups were recorded throughout the RPs.

Weeks 0 and 10 were dedicated to pre- and post-intervention strength tests. The actual RPs took place from weeks 1 to 9, following a linear periodization model in which volume decreases over time while intensity increases. Each session consisted of the following exercises in this specific order: leg press, chest press, seated row, leg extension, shoulder press, and lat pulldown. The exercises were performed on the same machines used for the tests (Biocircuit Wellness System, Technogym, Cesena, Italy).

Weeks 1 to 3 were used as introductive weeks for the RPE group, whereby the physiotherapists assigned the loads to the patients to ensure the correct RPE targets would be reached. Doing so allowed the patients to have time to practice with the RPE scale, as previous research showed that RPE accuracy could take up to three weeks [31].

After this familiarization period, starting from week 4 patients in the RPE group self-selected all the loads. Specifically, the patients were required to perform the prescribed sets and repetitions with a load that would correspond to the assigned RPE range, meanwhile the therapists would cue them showing the RPE scale based on RIR and the records of previous performance to aid the load selection. If the RPE score would fall outside of the target ranges, adjustments in load were made for the following sets [32]. Specifically, for every 1 RPE score above or below the target range, loads were decreased or increased by 4% to bring the RPE of subsequent sets closer to the prescribed range, as shown in Table 3.

If repetitions were missed due to excessive load, the set was considered a 10 RPE and the load was decreased by an additional 4% for every missed repetition, as shown in Table 4. Within a week, patients had three sessions with the same prescription (sets, repetitions, intensity) to gauge the RPEs correctly to the assigned repetitions. The repetition schemes (sets, repetitions) were the same for three consecutive weeks, as the intensity increased from one week to the other, so patients could learn how to increase and estimate the effort at a given repetition range. Every fourth week, the intensity remained constant and repetition schemes changed, so that patients could adapt to the new repetition ranges at the same intensity. Relative intensities in both groups were assigned based on the previously found relationship between loads, repetitions, and RPEs [24], as shown in Supplemental Table S1, so that the percentages of 1RMs in the %1RM group would correspond on average to the RPE ranges of the RPE group.

Rest periods were one minute for sets at RPE between 5 and 7, one minute and a half for sets at RPE between 6 and 8, and two minutes for sets at RPE between 7 and 9, as higher intensities require longer rest periods [27]. As for the strength tests, patients were allowed to use the Valsalva maneuver, given that they were required to train at high intensities and close to muscle failure. Patients were also required to complete at least two sessions per week, otherwise they would be excluded from the RPs.

### 2.6. Statistical Analysis

Statistical analysis was performed with SPSS statistical software package (version 20.0, IBM Corp., Armonk, NY, USA). Continuous variables are expressed as mean ± standard deviation (SD) and were compared with a *t*-test, paired for the within-group analysis, and unpaired for the between-group analysis. The assumption of normality was checked using the Shapiro–Wilk hypothesis test. Categorical variables are expressed as absolute and percentages values and were compared with the chi-square test. The level of statistical significance was set at *p* < 0.05.

Sample size was determined by feasibility and patients’ availability; hence, no formal a priori power analysis was performed [33]. Pre- and post-intervention variables with normal distribution were assessed using analysis of covariance (ANOVA), specifically, two-way repeated measures ANOVA considering time and load prescription method as predictive variables of strength. Within-group Cohen’s d effect size (ES) was calculated using the pooled pre- and post-intervention SD and 0.65 and 0.73 were identified as smallest effect size of interest (SESOI) for lower and upper body strength, respectively, based on Yamamoto et al.’s meta-analysis [34]. Between-group ES was calculated as the difference between groups’ mean change scores divided by the pooled SD of both groups’ change scores, as this is the appropriate method to compare results between two groups [35]. Based on the scale for determining the magnitude of ES in strength training research proposed by Rhea et al. [36], 0.5 was set as SESOI for the between groups comparison.

## 3. Results

### 3.1. Baseline Characteristics

Patient baseline anthropometric and clinical characteristics along with echocardiographic parameters are summarized in Table 5. Overall, both groups had similar baseline characteristics and no statistically significant differences were found except for basal heart rate, previous smoking habit, use of antiarrhythmics, and stroke volume.

### 3.2. Adherence and Adverse Effect Baseline Characteristics

Adherence to RPs was high with a mean of 24 completed sessions out of 27 (88%), with no statistically significant differences between the two groups (RPE group 88 ± 3% vs. %1RM group 89 ± 4%, *p* = 0.213). Furthermore, for the entire duration of the RPs, there were no cardiac alarm symptoms, i.e., shortness of breath, palpitation, chest pain, dizziness, or syncope, and no adverse CV events, such as exercise-induced malignant arrhythmias or cardiac ischemia.

### 3.3. Strength

Table 6 shows the groups’ pre-intervention 6RM strength test results for each exercise of the RPs. Baseline strength levels were similar between the two groups, with no statistically significant differences for any of the exercises. At the end of the RPs, significant increments in strength were recorded (ANOVA *p* < 0.05) in both groups compared to baseline results, except for the shoulder press in the %1RM group, with an increment approaching the level of significance (*p* = 0.053), as shown in Table 7. Within-group analysis showed ES greater than the SESOI on all the exercises for the RPE group, with relative percentage change between 19.1% and 24.7% (for the lat pulldown and the seated row respectively), while for the %1RM group, the ES for chest press, shoulder press, and lat pulldown were below the SESOI. Table 6 shows groups’ post-intervention 6RM strength test results for each exercise of the RPs. Overall, no statistically significant differences between the two groups were recorded (ANOVA *p* > 0.05). Between-group analysis demonstrated no advantages for one prescription method over the other, with ES below the SESOI. Patients’ individual change scores are shown in Supplementary Table S2.

### 3.4. RPE and Relative Intensity

**Figure 1:**
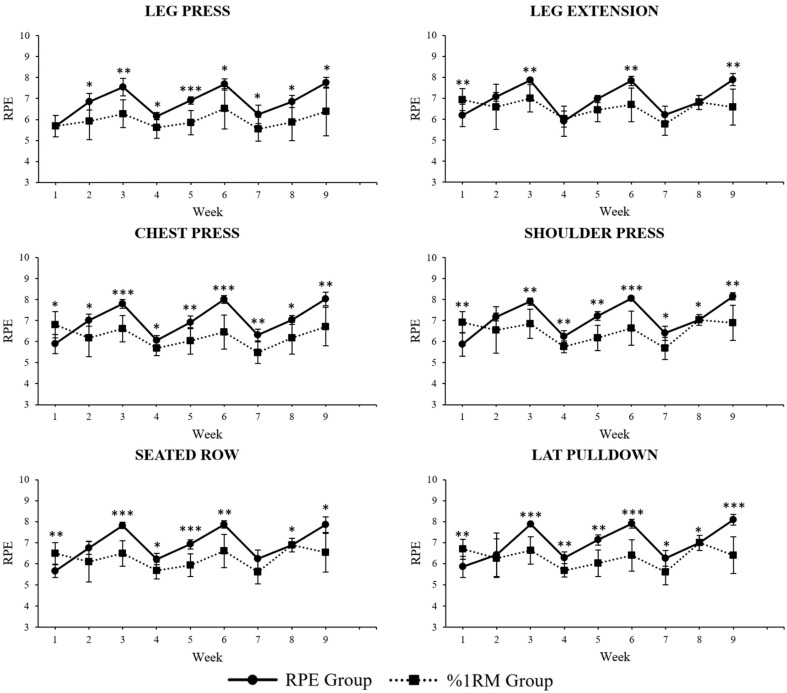
Weekly average RPE trends per exercise per group (RPE scores are shown in y-axis; comparisons made by two-way ANOVA); * *p* < 0.05, ** *p* < 0.01, *** *p* < 0.001.

Figure 1 shows weekly average RPE trends of all the exercises of the RPs for both groups throughout this study. Comparisons were made by two-way ANOVA. Specifically, for the leg press, the average RPE was higher in the RPE group from week 2 to 9; for the chest press, it was higher in the %1RM group in week 1, and then higher in the RPE group from week 2 to 9; for the seated row, it was higher in the %1RM group in week 1, and then higher in the RPE group from week 3 to 6 and in week 8 and 9; for the leg extension it, was lower in the %1RM group in week 1, and higher in the RPE group in week 3, 6, and 9; for the shoulder press, it was higher in the %1RM group in week 1 and then higher in the RPE group from week 3 to 9; for the lat pulldown, it was higher in the %1RM group in week 1 and then higher in the RPE group from week 3 to 9.

**Figure 2:**
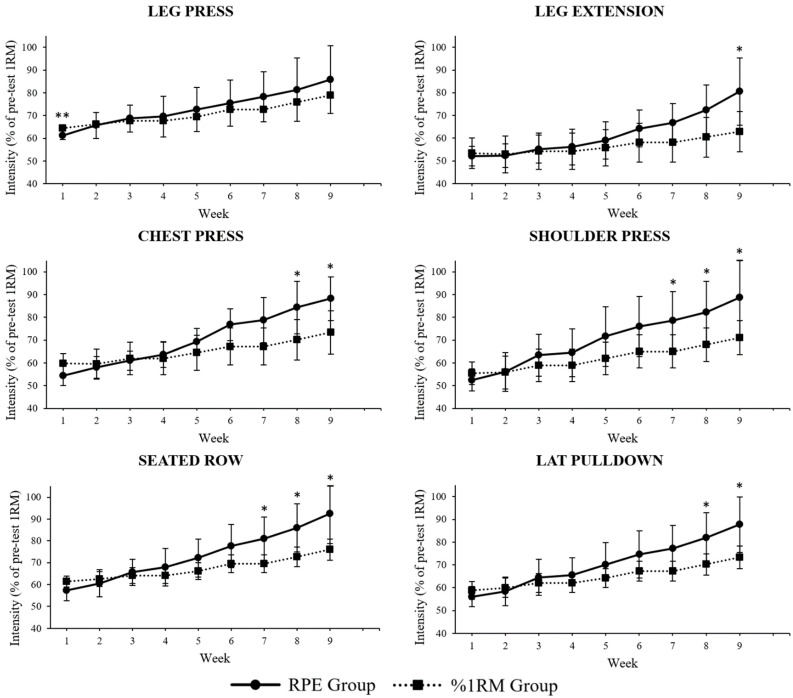
Weekly average relative intensity trends per exercise per group (comparisons made by two-way ANOVA); * *p* < 0.05, ** *p* < 0.01.

Figure 2 shows weekly average relative intensity trends of all the exercises of the RPs for both groups throughout this study. Relative intensity was defined as the load used in training divided by pre-intervention e1RMs. Specifically, for the leg press, the average relative intensity was higher in the %1RM group in week 1; for the chest press, it was higher in the RPE group in week 8 and 9; for the seated row, it was higher in the RPE group from week 7 to 9; for the leg extension, it was higher in the RPE group in week 9; for the shoulder press, it was higher in the RPE group from week 7 to 9; for the lat pulldown it, was higher in the RPE group in week 8 and 9.

## 4. Discussion

The purposes of this study were to assess the efficacy of the RPE based on RIR as prescription method for RT in CAD patients undergoing CR and to compare it to the standardized practice of percentage of 1RM. In line with our hypothesis, the recently developed RPE scale based on RIR proved to be an effective and practical tool for the prescription of RT intensity in CR settings. In fact, the RPE group showed statistically significant increases in strength across all the administered exercises of the RP, with relative percentage changes between 19.1% and 24.7%, and relevant ES compared to pre-intervention strength tests. Furthermore, RPE based on RIR demonstrated similar efficacy in terms of strength gains compared to percentage of 1RM prescription method, with post-intervention strength tests showing no statistically significant differences and trivial ES for all the exercises of the RPs between the two groups.

To our knowledge, this is the first study investigating the RPE based on RIR in clinical settings and outside the strength and conditioning research field [37]. The rationale to include RPE in training derives from the intrinsic variability of physical performance, which is subject to variations due to factors such as nutrition, sleep, and stressors, among others [38]. Daily fluctuations in performance can especially be observed in subjects with chronic conditions under multiple pharmacological treatments, such as CAD patients. These fluctuations can occur during initial strength tests, thereby affecting the results and consequently the training plan.

Moreover, even if the initial tests accurately reflect individual’s strength levels, they will not account for the progress or the rate at which they occur [39]. This can be observed in the relative intensity differences between the two groups towards the end of RPs, with statistically significant differences in week 7 to 9 for the shoulder press and the seated row, in week 8 and 9 for the chest press and the lat pulldown, and in week 8 for the leg extension. Patients in the RPE group likely trained at higher intensities during the last weeks because they were not limited by the pre-planned loads based on the initial strength tests, as they were allowed to increase the training intensity based on their current performance, while patients in the %1RM group may have trained with percentages not representative of their actual strength.

The higher relative intensities and RPE scores in the RPE group could also explain why this group saw statistically significant increases and relevant ES on all the exercises; meanwhile, the %1RM group saw no statistically significant increments in the shoulder press and only trivial ES for the chest press, shoulder press, and lat pulldown, despite no differences in training load (defines as sets × reps × load) being identified for the entire duration of this study. These results are in line with the current literature showing that RT programs with high loads are superior in inducing muscle strength gains when compared to low loads [40,41]. Also, despite the common practice of calculating the number of repetitions allowed at a given percentage of 1RM from textbook tables [29], these charts have recently been questioned [42], since there is high inter-individual variation in the number of repetitions that can be performed at the given percentage [43]. Therefore, considering all the variables affecting strength in clinical settings, prescribing RT intensity with RPE based on RIR could be more accurate than percentages in these scenarios, especially if, like the RP proposed in this study, multiple sets with a low number of repetitions and close to muscle failure are used, since previous research has demonstrated that these factors can improve the accuracy of RIR prediction [44]. Moreover, the two methods are not mutually exclusive and could be used in adjunctions for a more individualized prescription.

Since percentage-based loading can set unrealistic expectations for practitioners and patients alike, autoregulation can be implemented to overcome these limitations. Defined as the process of adjusting the training program over time based on individual’s current performance [45], autoregulation proved to be as effective as standardized percentage-base loading for strength improvements in a recent meta-analysis [27], and this study is the first exploring its applicability in clinical settings.

The present study has several limitations. Firstly, due to the limited availability of patients meeting the inclusion criteria during the time of recruitment, our analysis was limited to a small sample size; hence, larger clinical trials are necessary to confirm the current findings. Secondly, due to the high prevalence of CAD male subjects in cardiac rehabilitation facilities, the analyzed sample includes only men; therefore, it is not possible to extend the results of the current study to female subjects as well. Regarding the RPs, the percentages and the respective RPEs used for loads prescription are derived from e1RMs and not actual measured 1RMs, since 1RM testing would have been impractical in patients uncustomed to RT. Moreover, the relationship between loads, repetitions, and RPEs, used to develop the RPs and to ensure the patients would train at the same intensities, is based on a single study conducted on healthy young subjects performing the barbell back squat [24]. Thus, it is possible the same relationship does not subsist in CAD patients performing different exercises. Other potential limitations of this study could be if practitioners administrating exercise are not experienced with the RPE scale based on RIR and if the patients are not given a period of practice with this scale, like the introductive weeks of the RP. Uncontrolled diet represents another variable that needs to be considered, since calories and protein intakes are major contributors of skeletal muscle hypertrophy, which is closely related to muscle strength. Lastly, QoL assessments and measurements of cardiorespiratory fitness were not conducted to focus the analysis on the training variables and the individual results.

## 5. Conclusions

In this study, the novel RPE scale based on RIR proved to be an effective method for the prescription of RT exercise in CR of CAD patients, showing similar efficacy to the standardized practice of percentages of 1RM-based training. Further research is needed to confirm and extend the results of the present study.

## Figures and Tables

**Table 1:** Resistance training rate of perceived exertion scale based on number of repetitions on reserve.

Rate of Perceived Exertion	
Score	Description of Perceived Exertion
10	No further repetitions could be performed
9.5	No further repetitions but load could be increased
9	One more repetition could be performed
8.5	One to two repetitions could be performed
8	Two more repetitions could be performed
7.5	Two to three repetitions could be performed
7	Thee more repetitions could be performed
6.5	Three to four repetitions could be performed
6	Four more repetitions could be performed
5.5	Four to five repetitions could be performed
5	Five more repetitions could be performed

Values in the score column correspond to the perceived level of exertions reported by the subject in the description column.

**Table 2:** Rehabilitation protocol.

Week	RPE Group	%1RM Group
0	6RM tests	6RM tests
1	3 × 10@5–7 RPE	3 × 10@65%
2	3 × 10@6–8 RPE	3 × 10@66.5%
3	3 × 10@7–9 RPE	3 × 10@68%
4	3 × 8@5–7 RPE	3 × 8@68%
5	3 × 8@6–8 RPE	3 × 8@70%
6	3 × 8@7–9 RPE	3 × 8@73%
7	4 × 6@5–7 RPE	4 × 6@73%
8	4 × 6@6–8 RPE	4 × 6@76%
9	4 × 6@7–9 RPE	4 × 6@79%
10	6RM tests	6RM tests

Values are displayed as sets × repetitions × load (RPE or percentage of 1RM).

**Table 3:** Examples of load adjustment for target RPE.

Reported RPE	Target RPE 6–8
4	Increase load by 8%
5	Increase load by 4%
6	No modification
7	No modification
8	No modification
9	Decrease load by 4%
10	Decrease load by 8%

Load adjustments in case of a reported RPE outside of the target RPE range between 6 and 8.

**Table 4:** Examples of load adjustment for missed repetitions.

Protocol	Repetitions Performed	Load Adjustment
6@7–9 RPE	5	Decrease load by 8%
6@6–8 RPE	5	Decrease load by 12%
6@5–7 RPE	5	Decrease load by 16%

Load adjustments in case of one missed repetition to three different protocols.

**Table 5:** Patient baseline characteristics.

Characteristics	Population (*n* = 16)	RPE Group (*n* = 8)	%1RM Group (*n* = 8)	*p*
Age (years)	60 ± 8	61 ± 9	59 ± 7	0.661
BMI (kg/m^2^)	26 ± 2	26 ± 2	26 ± 1	0.838
HR (bpm)	67 ± 7	63 ± 8	71 ± 4	0.031 *
SBP (mmHg)	118 ± 9	123 ± 9	26 ± 2	0.715
DBP (mmHg)	74 ± 9	75 ± 5	75 ± 5	0.122
ACS, *n* (%)	12 (75)	6 (50)	6 (50)	-
PTCA/CABGs, *n* (%)	7 (44)	5 (63)	6 (75)	0.262
Systemic arterial hypertension, *n* (%)	8 (50)	5 (63)	3 (38)	0.480
Type 2 Diabetes, *n* (%)	1 (6)	1 (13)	0 (0)	0.401
Smoking habits, *n* (%)	7 (44)	5 (63)	2 (25)	0.023 *
Family history of CVD, *n* (%)	5 (31)	3 (38)	2 (25)	0.480
Beta blockers, *n* (%)	14 (88)	8 (100)	6 (75)	0.325
ACEi/ARB, *n* (%)	14 (88)	8 (100)	6 (75)	0.325
CA, *n* (%)	2 (13)	2 (25)	0 (0)	0.143
MRA, *n* (%)	0 (0)	0 (0)	0 (0)	-
Diuretics, *n* (%)	3 (19)	2 (25)	1 (13)	0.223
Statins, *n* (%)	16 (100)	8 (100)	8 (100)	-
Antiarrhythmics, *n* (%)	4 (25)	1 (13)	3 (38)	0.034 *
EF (%)	54 ± 6	52 ± 6	56 ± 6	0.187
GLS (%)	−17.4 ± 4.1	−17.5 ± 3.9	−17.3 ± 4.5	0.942
SV (mL)	65.0 ± 12.7	58.8 ± 7.0	71.2 ± 14.4	0.046 *
LAVi (mL/m^2^)	31.2 ± 7.6	27.8 ± 6.5	34.6 ± 7.4	0.07
E/A	1.1 ± 0.3	1.0 ± 0.2	1.2 ± 0.3	0.334
E/e’	6.0 ± 1.6	5.4 ± 1.5	6.7 ± 1.5	0.103

BMI: body mass index; HR: heart rate; SBP: systolic blood pressure; DBP: diastolic blood pressure; ACS: acute coronary syndrome; PTCA: percutaneous transluminal coronary angioplasty, CABGs: coronary artery bypass graft surgery; CVD: cardiovascular disease; ACEi: angiotensin-converting enzyme inhibitors; ARB: angiotensin receptor blockers; CA: calcium antagonists; MRA: mineralocorticoid receptor antagonists; EF: ejection fraction; GLS: global longitudinal strain; SV: stroke volume; LAVi: left atrial volume index; * *p* < 0.05.

**Table 6:** Pre- and post-intervention 6RM strength test results.

Exercise	RPE Group	%1RM Group	*p*	RPE Group	%1RM Group	*p*	ES
	Pre-Intervention Strength Test			Post-Intervention Strength Test			
Leg press	121.9 ± 32.2	120.6 ± 23.4	0.930	146.1 ± 36.1	145.3 ± 24.4	0.955	−0.03
Chest press	37.7 ± 10.5	37.9 ± 13.9	0.968	44.9 ± 8.8	45.1 ± 12.9	0.965	0.00
Seated row	56.3 ± 18.7	59.13 ± 12.21	0.721	70.1 ± 17.3	72.4 ± 11.3	0.763	0.10
Leg extension	62.9 ± 18.1	66.13 ± 14.0	0.694	77.1 ± 19.3	82.4 ± 16.2	0.565	−0.29
Shoulder press	28.6 ± 6.9	28.88 ± 6.6	0.942	34.4 ± 7.4	33.8 ± 7.4	0.868	0.18
Lat pulldown	39.3 ± 10.1	41.0 ± 9.0	0.719	46.8 ± 10.3	48.4 ± 10.8	0.780	0.05

Values in RPE and %1RM group columns are expressed in kg as mean ± standard deviation (comparisons made by two-way ANOVA); ES: between-group effect size.

**Table 7:** Strength variation per exercise per group.

Strength Improvements								
	RPE Group				%1RM Group			
Exercise	Change Score (95% CI)	%	*p*	ES	Change Score (95% CI)	%	*p*	ES
Leg press	24.3 (12.4–36.1)	19.9	0.005 *	0.71 ^#^	24.6 (16.2–33.1)	20.4	0.001 *	1.03 ^#^
Chest press	7.3 (4.9–9.6)	19.3	0.001 *	0.75 ^#^	7.3 (1.9–12.6)	19.1	0.034 *	0.54
Seated row	13.9 (8.6–19.1)	24.7	0.001 *	0.77 ^#^	13.3 (9.9–16.6)	22.4	0.000 *	1.13 ^#^
Leg extension	14.3 (11.1–17.4)	22.7	0.000 *	0.76 ^#^	16.3 (9.9–22.6)	24.6	0.002 *	1.08 ^#^
Shoulder press	5.8 (2.9–8.6)	20.1	0.005 *	0.80 ^#^	4.9 (0.8–9.0)	16.9	0.053	0.70
Lat pulldown	7.5 (4.3–10.7)	19.1	0.003 *	0.74 ^#^	7.3 (3.1–11.4)	17.7	0.011 *	0.73

Values in change score columns are expressed in kg and 95% confidence intervals (comparisons made by two-way ANOVA); ES: within-group effect size; * *p* < 0.05; ^#^ non-trivial ES.

## Data Availability

The data presented in this study are available on request from the corresponding authors.

## Supplementary Materials

The following supporting information can be downloaded at: https://www.mdpi.com/article/10.3390/jcdd12010008/s1, Table S1: Relationship between percentage of 1RM, repetitions and RPE; Table S2: Patients’ individual change score per exercise per group.
