# Supplementary material for: Rate of Perceived Exertion Based on Repetitions in Reserve Versus Percentage of One-Repetition Maximum for Resistance Training Prescription in Cardiac Rehabilitation: A Pilot Study
Source: J Cardiovasc Dev Dis. 2024 Dec 27;12(1):8. doi: 10.3390/jcdd12010008 (PMC11766398; doi:10.3390/jcdd12010008)
Supplement: Supplementary file 1 [file jcdd-12-00008-s001.zip › jcdd-3327244-supplementary.pdf]

Supplementary Table S1 Relationship between percentage of 1RM. repetitions and RPE.

		REPETITIONS PERFORMED									
		**1**	**2**	**3**	**4**	**5**	**6**	**7**	**8**	**9**	**10**
RATE OF PERCEIVED EXERTION	**10**	100%	95.0%	91.0%	87.0%	85.0%	83.0%	81.0%	79.0%	76.0%	73.0%
	**9.5**	97.0%	93.0%	89.0%	86.0%	84.0%	82.0%	80.0%	77.5%	74.5%	71.5%
	**9**	95.0%	91.0%	87.0%	85.0%	83.0%	81.0%	79.0%	76.0%	73.0%	70.0%
	**8.5**	93.0%	89.0%	86.0%	84.0%	82.0%	80.0%	77.5%	74.5%	71.5%	69.1%
	**8**	91.0%	87.0%	85.0%	83.0%	81.0%	79.0%	76.0%	73.0%	70.0%	68.2%
	**7.5**	89.0%	86.0%	84.0%	82.0%	80.0%	77.5%	74.5%	71.5%	69.1%	67.4%
	**7**	87.0%	85.0%	83.0%	81.0%	79.0%	76.0%	73.0%	70.0%	68.2%	66.6%
	**6.5**	86.0%	84.0%	82.0%	80.0%	77.5%	74.5%	71.5%	69.1%	67.4%	65.8%
	**6**	85.0%	83.0%	81.0%	79.0%	76.0%	73.0%	70.0%	68.2%	66.6%	65.0%
	**5.5**	84.0%	82.0%	80.0%	77.5%	74.5%	71.5%	69.1%	67.4%	65.8%	64.2%
	**5**	83.0%	81.0%	79.0%	76.0%	73.0%	70.0%	68.2%	66.6%	65.0%	63.5%

Supplementary Table S2 Patients' individual change scores per exercise per group.

	INDIVIDUAL CHANGE SCORE											
	**Leg press**		**Chest press**		**Seated row**		**Leg extension**		**Shoulder press**		**Lat pulldown**	
**Patient**	**RPE Group**	**%1RM Group**	**RPE Group**	**%1RM Group**	**RPE Group**	**%1RM Group**	**RPE Group**	**%1RM Group**	**RPE Group**	**%1RM Group**	**RPE Group**	**%1RM Group**
1	10	15	6	-10	10	15	6	14	1	-7	4	-2
2	55	32	14	7	25	10	17	11	10	4	10	5
3	38	5	4	10	5	7	15	4	8	0	13	0
4	15	45	4	5	15	10	10	20	6	10	6	16
5	10	30	8	10	18	20	13	35	1	8	6	8
6	35	30	7	7	8	14	18	11	12	5	14	11
7	24	20	10	14	23	10	20	15	5	9	7	10
8	7	20	5	15	7	20	15	20	3	10	0	10

Values in the exercise columns are expressed in kg
